# Supplementary material for: WordCommentsAnalyzer: A windows software tool for qualitative research
Source: F1000Res. 2018 Sep 4;7:536. Originally published 2018 May 3. [Version 2] doi: 10.12688/f1000research.14819.2 (PMC6137411; doi:10.12688/f1000research.14819.2)
Supplement: Supplementary file 3 [file f1000research-7-17614-s0002.tgz › b2a79496-4ada-47c4-9682-a99ce867836d.zip!/Eight Iranian Health Journals/Journal of Health System Research _29.docx]

© Journal of Health System Research . This work is licensed under CC BY-NC 4.0 (https://creativecommons.org/licenses/by-nc/4.0/).

Volume & Issue: دوره 9، شماره 3؛ 1392

Title: بررسي تأثير ورزش بر اشتها و ميزان سرمي هورمون گرلين: پيشنهادهايي براي كنترل وزن

http://hsr.mui.ac.ir/index.php/jhsr/article/view/693

مقدمه: گرلين (Ghrelin) تنها هورمون شناخته شده مؤثر در افزايش اشتها در انسان می‌باشد و ممكن است به عنوان عامل مؤثری در افزايش اشتها و دريافت غذا پس از كاهش وزن عمل كند. كشف راه‌‌حل‌هايي براي كاهش گرلين و كاهش اشتها جهت حفظ كاهش وزن مورد نياز است. آگاهي در مورد رابطه بين ورزش و اشتها هم براي ورزشكاران و هم براي افرادي كه علاقه‌مند به حفظ وزن مناسب هستند، اهميت دارد. هدف از اين مقاله مروري، بررسي شواهد موجود در مورد رابطه بين گرلين و عوامل مربوط به شيوه زندگي و همچنين ارایه پيشنهادهايي براي كنترل وزن بود. روش‌ها: مطالعه حاضر، يك مطالعه مروري بود. با استفاده از بانك‌هاي اطلاعاتي و موتورهاي جستجوي علمي مانند PubMed و Elsevier و كليد واژه‌های ورزش، اشتها، گرلين، چاقي و كنترل وزن مقالات با طرح‌هاي كارآزمايي باليني انتخاب شدند. بازه زماني مقالات مورد استفاده از سال 2000 تا سال 2012 ميلادي بود. يافته‌ها: مطالعات نشان دادند، ورزش‌هاي طولاني مدت باعث افزايش جزیي در دريافت انرژي مي‌شود كه شايد به علت تغيير در ميزان هورمون‌هاي تنظيم كننده اشتها باشد. شواهد موجود نشان مي‌دهد كه ورزش‌هاي شديد باعث بي‌اشتهايي كوتاه مدت بعد از ورزش مي‌گردد و تحت عنوان بي‌اشتهايي بعد از ورزش (Exercise-induced anorexia) ناميده مي‌شود، ولي مكانيسم اين تأثير هنوز شناخته نشده است. نتيجه‌گيري: جهت كاهش وزن مطلوب، ورزش در دوره پس از سرو غذا (Postprandial period) ممكن است اثر سيري غذا را براي مدت زمان بيشتري تداوم بخشد. ورزش‌هاي طولاني مدت ممكن است باعث افزايش جزیي در دريافت انرژي به علت افزايش هورمون گرلين شوند، اما در تمام شواهد ذكر شده نياز به تحقيقات بيشتري جهت تأييد قطعي آن‌ها مي‌باشد.

Title: لينولئيك اسيد كنژوگه و سندرم متابوليك: مرور سيستماتيك

http://hsr.mui.ac.ir/index.php/jhsr/article/view/735

مقدمه: اگرچه بسیاری از مطالعات حیوانی نشان داده‌اند که لینولئیک اسید کنژوگه می‌تواند در جلوگیری از سندرم متابولیک نقش مهمی ایفا کند، اما مطالعات انسانی در این زمینه بسیار کم و نتایج حاصل از آن‌ها بسیار متناقض است. این مطالعه با هدف خلاصه کردن اطلاعات مربوط به مطالعات حیوانی و کارآزمایی بالینی در رابطه با تأثیر ایزومرهای مختلف لینولئیک اسید کنژوگه بر اجزای سندرم متابولیک انجام گردید. روش‌ها: پايگاه اطلاعاتي PubMed و ISI از ژانويه سال 2000 تا فوريه سال 2012 مورد جستجو قرار گرفت. مقالات كارآزمايي باليني و آزمايشگاهي که به بررسي تأثير لينولئيك اسيد كنژوگه تركيبي و يا هر كدام از ايزومرهاي لينولئيك اسيد كنژوگه بر مقاومت به انسولين، چاقي، ديس ليپيدمي و فشار خون پرداخته بودند، انتخاب شدند. از 320 مطالعه یافت شده در این زمینه، 24 مطالعه که دارای شرایط ورود بودند انتخاب شدند. یافته‌ها: مطالعات حیوانی نشان داده‌اند که لینولئیک اسید کنژوگه خصوصاً ایزومر 9 سیس 11 ترانس، می‌تواند سبب بهبود مقاومت به انسولین، کاهش کلسترول و تری‌گلیسیرید سرم گردد. در حالی‌که مطالعات کارآزمایی بالینی نتوانستند تأثیر لینولئیک اسید کنژوگه را بر مقاومت به انسولین و سطح لیپیدهای سرم در انسان را نشان دهند. نتیجه‌گیری: به طور کلی ایزومرهای مختلف لینولئیک اسید کنژوگه دارای اثرات متفاوتی می‌باشند و می‌توان اثرات کاهش مقاومت به انسولین و کاهش چربی بدن را به ایزومر 9 سیس 11 ترانس و کاهش فشار خون را به ایزومر 10 ترانس 12 سیس لینولئیک اسید کنژوگه نسبت داد.

Title: تأثیر کم‌خوابی بر وزن بدن؛ مروری بر شواهد موجود

http://hsr.mui.ac.ir/index.php/jhsr/article/view/751

مقدمه: چاقي يک بيماري مزمن چند عليتي پيچيده است که شايع‌ترين اختلال متابوليسم چربي در انسان مي‌باشد و ناشي از تعامل عوامل ژنتيکي و محيطي است. با توجه به روند سریع رو به رشد چاقی، به نظر عوامل محیطی نقش مهم‌تری نسبت به عوامل ژنتیکی دارند. از بین عوامل محیطی می‌توان به دریافت‌ها و عادات غذایی، سطح فعالیت بدنی و الگو و مدت زمان خواب افراد اشاره کرد. مطالعاتی که تاکنون انجام شده‌اند، بیشتر به بررسی انرژی دریافتی افراد و سطح فعالیت بدنی افراد و تعادل بین آن دو پرداخته‌اند. با این حال الگو و مدت زمان خواب افراد از دیگر عوامل تأثیرگذار بر سیستم‌های فیزیولوژیکی است که کمتر مورد بررسی قرار گرفته است. در مطالعه مروری حاضر، به بررسی یافته‌های مطالعاتی که ارتباط مدت زمان خواب و وزن بدن را در بین بزرگسالان و نوجوانان بررسی کرده‌اند، به تفکیک خواهیم پرداخت. روش‌ها: با استفاده از سیستم موتور جستجوی Pubmed مطالعاتی با طراحی‌های مختلف (مقطعی و Cohort) که در بین سال‌های 2012-1990 چاپ شده بودند، بررسی شدند. یافته‌ها: خطر ابتلا به چاقی در بزرگسالانی که کمتر از 6 ساعت می‌خوابند بیشتر بود. همچنین اکثر مطالعات مقطعی در کودکان نیز نشان داده‌اند که کم‌خوابی با افزایش خطر ابتلا به چاقی همراه است. مطالعاتی که نشان داده‌اند کم‌خوابی با کاهش وزن همراه است بیشتر در بزرگسالان مسن‌تر انجام شده‌اند. محدودیت اصلی که در تمام این مطالعات به چشم می‌خورد، نامشخص بودن علت کم‌خوابی است که ممکن است به علت وقت ناکافی و یا اختلالات خواب باشد. نتیجه‌گیری: به نظر می‌رسد کم‌خوابی با افزایش وزن همراه باشد که این رابطه در بزرگسالان جوان و کودکان بیشتر مشهود می‌باشد.

Title: بررسی نقش اخلاق بر سلامت روان در اجتماع از دیدگاه نهج البلاغه

http://hsr.mui.ac.ir/index.php/jhsr/article/view/768

مقدمه: يكي از عوامل موثر در ايجاد آرامش ، ایمان و اخلاق نیکو و شایسته است؛ زیرا احساس شاد ی به انسان دست داده و باعث نشاط و سلامت روانی مى شود و به دنبال آن سلامت فيزيكي تامین و انسان را به فعاليت هاى صحيح زندگى وادار مى كند. یکی از والاترین اهداف دین اسلام، تکامل مکارم اخلاقی است ،تا آنجا که پیامبر اکرم(ص) می فرمایند: «اِنّی بُعثتَ لا تَتَمّم مَکارمَ الاَخلاق» . اخلاق پسندیده با زدودن کینه ها و جذب قلب ها، زمینه روابط اجتماعی مناسب و آرامش را فراهم می آورد. حضرت امام علی(ع) می فرمایند: «هر کس اخلاقش نیکو باشد، راه های زندگی برایش هموار می گردد.» لذا پژوهش حاضر با هدف بررسی نقش اخلاق بر سلامت روان از دیدگاه امام علی (ع) انجام گرفت . روش : پژوهش حاضر یک مطالعه کتابخانه ای است که نقش اخلاق پسندیده بر سلامت روان در کلام امام علی (ع) بررسی شده است و در آن سعی شده از قرآن و نهج البلاغه وغررالحکم استتفاده گردد. در ابتدا اخلاق تعریف و سپس صفات پسندیده اخلاقی در کلام امام علی (ع)ذکر شده و آثار هر کدام بر سلامت روان بیان گردیده است . یافته ها : دراین مطاله ، نقش اخلاق بر سلامت روان از دیدگاه امام علی (ع) مورد بررسی قرار گرفت و یافته های تحقیق نشان داد حسن خلق از موارد مهم اثر گزار بر سلامت روان است و صفات  پسنذیده در ارتقای آن موثر است.  بحث و نتیجه گیری :اخلاق عبارت است از یک سلسله خصلتها و سجایا و ملکات اکتسابی که بشر آن را به عنوان اصول اخلاق می پذیرد.با یاد خدا و اخلاق نیکو ،انسان به آرامش و سلامت روان دست می یابد. پیامبر اکرم (ص) محبوب ترین فرد  نزد خداوند را خوش خلق ترین مردم می دانند و حضرت علی (ع) حسن خلق را سرلوحه ویژگی های مومن می دانند. حسن خلق، تواضع، بردباري و شرح صدر، عفو و گذشت و مدارا كردن، خوش گماني ، صله رحم ، تغافل، رازداري و انصاف از جمله صفات مناسب اخلاقي است كه به بهبود روابط اجتمایی و سلامت روان كمك مي كند.

Title: اسیدهای چرب امگا 3 و کبد چرب غیر الکلی

http://hsr.mui.ac.ir/index.php/jhsr/article/view/687

مقدمه: اسيدهاي چرب امگا 3 داراي اثرات مفيد ثابت شده‌اي براي بيماري‌هاي قلبي- عروقي و چربی خون بالا (Hyperlipidemia) است. به همين دليل استفاده از مكمل امگا 3 براي بيماران مبتلا به كبد چرب غير الكلي پيشنهاد شده است. هدف از اين مطالعه، مرور مطالعاتي بودند كه ارتباط بين مصرف امگا 3 و كبد چرب غير الكلي را بررسي كرده‌اند. روش‌ها: در مطالعه حاضر در پایگاه اطلاعاتی PubMed واژه‌هاي كبد چرب غير الكلي، اسيدهاي چرب امگا 3، دوكوزاهگزانوئيك اسيد و ايكوزاپنتانوئيك اسيد جستجو شد. در اين جستجو 15 مطالعه در مورد كبد چرب غير الكلي و امگا 3 انجام شده بود. در بین اين 15 مطالعه، 4 مطالعه به طور كامل در دسترس قرار نداشت و سه مطالعه نيز از نظر طراحي قوي نبود و در پايان 8 مطالعه انتخاب شد. يافته‌ها: امگا3 يك تنظيم كننده بيان ژن در كبد است. مطالعات اثبات كردند که مصرف امگا 3 باعث كاهش استئاتوزيس كبدي، بهبود حساسيت انسوليني و كاهش ماركرهاي التهاب مي‌شود. كارآزمايي‌هاي باليني دیگری بايد براي تأييد اين يافته‌ها انجام شود. نتيجه‌گيري: مصرف امگا 3 باعث بهبود علايم مربوط به كبد چرب غير الكلي مي‌گردد، اما مطالعات بيشتری بايد در اين زمينه انجام شود.

Title: بررسی عوامل بازدارنده انجام پژوهش از دیدگاه اعضای هیأت علمی و ارایه طرحی برای مقابله با این عوامل در دانشگاه علوم پزشکی رفسنجان

http://hsr.mui.ac.ir/index.php/jhsr/article/view/481

مقدمه: توليدات علمي به ويژه مقالات پژوهشی اهميت بسزايي در توسعه علمي كشورها دارند. مطالعه حاضر با هدف، تعیین عوامل بازدارنده انجام پژوهش از دیدگاه اعضای هیأت علمی و ارایه طرحی برای مقابله با این عوامل در دانشگاه علوم پزشکی رفسنجان انجام شد. روش‌ها: مطالعه توصیفی- مقطعی حاضر در سال 1388 انجام شد. ابزار جمع‌آوری اطلاعات، پرسش‌نامه طراحی شده دو قسمتی شامل سؤالات دموگرافیک و سؤالاتی در خصوص موانع انجام تحقیق بر اساس پنج محور اقتصادی و مالی، سازمانی و اداری، شخصی و اجتماعی، ‌‌حرفه‌ای و تخصصی، وسایل و امکانات بود. اطلاعات با استفاده از آزمون‌های آماری توصیفی مورد تجزیه و تحلیل قرار گرفت. یافته‌ها: در مجموع 61 نفر از اعضای هيأت علمي دانشگاه پرسش‌نامه‌ها را تکمیل نمودند (میزان پاسخگویی 5/53 درصد). حداقل و حداکثر سن شرکت‌کنندگان به ترتیب 27 و 57 سال و ميانگين سنی آنان 85/6 ± 24/40 سال بود. 41 نفر از شركت ‌كنندگان (49/69 درصد) را مردان تشكيل مي‌دادند. موانع سازمانی و اداری، شخصی و اجتماعی و اقتصادی و مالی به ترتیب مهم‌ترین موانع تحقیق ذکر شده بودند. نتيجه‌گيري: به منظور رفع موانع مورد نظر اعضای هيأت علمي، باید درخواست‌هاي آنان را در سه سطح دانشگاه و وزارتخانه، امور پژوهشي دانشگاه و دانشكده‌ها و درخواست‌هاي ويژه مورد پيگيري قرار داد.

Title: مقایسه روش‌های پواسن کریگینگ نقطه‌ای و بیز تجربی در تنظیم نقشه‌ میزان بروز سرطان گوارش در ایران

http://hsr.mui.ac.ir/index.php/jhsr/article/view/596

مقدمه: نقشه‌بندی میزان رویداد یک بیماری یا مرگ و میر ناشی از بیماری‌های مختلف بر روی نقشه جغرافیایی یکی از موضوعات مورد علاقه متخصصان و برنامه‌ریزان امور بهداشتی به ویژه اپیدمیولوژیست‌ها می‌باشد. از جمله روش‌های برآورد پارامتر، نقشه روش بیز تجربی و روش پواسن کریگینگ نقطه‌ای می‌باشد. این مطالعه به منظور به کارگیری این دو روش در نقشه‌بندی میزان بروز سرطان گوارش در ایران و مقایسه‌ دقت (واریانس) آن‌ها انجام شد. روش‌ها: در مطالعه حاضر که از نوع کاربردی/ بوم‌شناسی بود از داده‌های ثبت شده توسط اداره سرطان مرکز مدیریت بیماری‌های غیر واگیر وزارت بهداشت، درمان و آموزش پزشکی استفاده شد. داده‌هایی که در سطح 336 شهرستان و در طول سال‌های 86-1382 جمع‌آوری شده بود، مورد تجزیه و تحلیل قرار گرفت. نرم‌افزارهای  Open Bugsو Space Stat برای برآورد پارامترها و نرم‌افزار ArcGIS 9.3 برای نمایش برآوردها بر روی نقشه به کار گرفته شدند. یافته‌ها: میانگین میزان بروز بر اساس روش بیز تجربی 99/12 و با روش پواسن کریگینگ نقطه‌ای، 64/12 برآورد شدند. میانگین واریانس میزان بروز با روش بیز تجربی 83/2 و با روش پواسن کریگینگ نقطه‌ای، 85/3 به دست آمد. بیشترین میزان بروز بر اساس روش بیز تجربی 23/42 با واریانس 16/2 مربوط به شهرستان ساری و کمترین میزان بروز 48/0 با واریانس 08/0 مربوط به شهرستان سرباز و بیشترین برآورد میزان بروز با روش پواسن کریگینگ 35/40 با واریانس 79/0 مربوط به شهرستان ساری و کمترین میزان بروز 30/0 با واریانس 54/2 مربوط به شهرستان سرباز محاسبه شدند. روش پواسن کریگینگ نقطه‌ای با دامنه‌ واریانس کمتری پارامترها را برآورد می‌کند. نتیجه‌گیری: پیشنهاد می‌شود از روش پواسن کریگینگ نقطه‌ای در برآورد پارامترهای نقشه‌بندی بیماری استفاده شود؛ چرا که از هموار سازی کمتر و دقت بیشتری برخوردار است.

Title: ارزیابی مواجهه با صداهای تراز فشار صوت بالا بر عملکرد و میزان خطای انسان در انجام فعالیت‌های دستی

http://hsr.mui.ac.ir/index.php/jhsr/article/view/684

مقدمه: صوت ناخواسته به عنوان يكي از مهم‌ترين عوامل فيزيكي محيط‌هاي كار در اكثر واحدهاي توليدي مطرح است و مشكلات بسيار زيادي را براي كارگران صنايع ايجاد مي‌نمايد. صدا را می‌توان به عنوان یکی از عوامل محیطی دانست که دارای اثرات جسمی و روانی بر انسان می‌باشد. این مطالعه با هدف، تعیین اثرات مواجهه با صدا بر عملکرد جسمی و تلفیق آن با عملکرد ذهنی انسان انجام شد. مواد و روش‌ها: مطالعه حاضر در فشار صوت‌های 70، 90 و 110 دسی‌بل A و ایجاد صدای سفید با دو عامل مشخصات فیزیکی و ایجاد حالات مختلف منبع فشار صوت، تأثیر صدا را بر عملکرد 50 نفر از دانشجویان دانشگاه علوم پزشکی شیراز (25 مرد و 25 زن) با انجام آزمون هماهنگی دو دست سنجید. یافته‌ها: در این مطالعه نشان داده شد که جنسیت (مرد و زن) و حالت‌های ایجاد منبع فشار صوت در زمان عملکرد اختلاف معنی‌داری از نظر آماری ندارند (05/0 < P) و زمان عملکرد با افزایش فشار صوت افزایش پیدا می‌کند که به دنبال آن عملکرد کاهش می‌یابد و از نظر آماری عملکرد بین 70 و 90 دسی‌بل اختلاف معنی‌داری را نشان نمی‌دهند. عملکرد در 110 دسی‌بل با عملکرد در هر یک از فشار صوت‌های 70 (05/0 > P) و 90 دسی‌بل (001/0 > P) اختلاف معنی‌داری دارد. نتیجه‌گیری: به طور کلی عملکرد با افزایش فشار صوت کاهش می‌یابد و به دنبال آن خطای فرد افزایش چشمگیری را نشان می‌دهد.

Title: بررسی وضعیت سلامت روان و میزان مواجهه با رویدادهای استرس‌زای زندگی در پرستاران شاغل در اورژانس 115

http://hsr.mui.ac.ir/index.php/jhsr/article/view/689

مقدمه: استرس‌های رواني محيط كار و زندگی بر سلامت رواني پرستاران تأثير مي‌گذارد و مي‌تواند به كاركرد شغلي و ايمني آنان و کاربران مراکز ارایه خدمات بهداشتي آسيب وارد كند. از این رو مطالعه حاضر با هدف، بررسی وضعیت سلامت روان و میزان مواجهه با رویدادهای استرس‌زای زندگی در پرستاران شاغل در خدمات اورژانس تهران انجام شد. روش‌ها: مطالعه مقطعی– توصيفي حاضر بر روی 200 نفر از پرستاران شاغل در خدمات اورژانس تهران که به صورت تصادفی ساده انتخاب شده بودند، انجام شد. برای سنجش سلامت روان از پرسش‌نامه سلامت عمومی (General health questionnaire یا GHQ) و جهت سنجش رويدادهاي زندگي از پرسش‌نامه رويداد زندگي Pickle (Pickle's life event questionnaire) استفاده شد. پس از تکمیل پرسش‌نامه‌ها، اطلاعات این مطالعه به وسیله نرم‌افزار SPSS نسخه 16 و با استفاده از آزمون 2c و در سطح معنی‌داری 05/0 مورد تجزیه و تحلیل قرار گرفت. یافته‌ها: رویدادهای زندگی با سلامت روان ارتباط معنی‌داری داشت (001/0 > P). بر اساس یافته‌های پژوهش، افراد دارای مدرک کارشناسی پرستاری نسبت به تکنسین‌های اتاق عمل و هوشبری از سلامت روان بهتری برخوردار بودند (001/0 > P) و رویدادهای استرس‌زای کمتری تجربه کرده بودند (001/0 > P). نتیجه‌گیری: پیشنهاد می‌شود برنامه‌های منظم و مدون آموزشی و مشاوره روان‌شناسی برای این افراد انجام شود و با گسترش حمایت‌های اجتماعی و خانوادگی سطح سلامت این قشر را افزایش داد تا از این طریق ارایه خدمات به مددجویان نیز ارتقا یابد.

Title: پیشگویی مصرف سیگار در نوجوانان: ارزیابی هویت خود سیگار کشیدن در نظریه رفتار برنامه‌ریزی شده

http://hsr.mui.ac.ir/index.php/jhsr/article/view/701

مقدمه: نوجوانی زمان شروع مصرف سیگار است و شناسایی پیشگویی کننده‌های مصرف برای اجرای برنامه‌های کنترل مصرف دخانیات ضروری می‌باشد. مطالعه حاضر با هدف، تعیین پیشگویی کننده‌های مصرف سیگار در نوجوانان و با ارزیابی نقش هویت خود سیگار کشیدن در نظریه رفتار برنامه‌ریزی شده انجام گرفت. روش‌ها: در مطالعه مقطعی حاضر، 250 دانش‌آموز پسر دبیرستانی شهرستان زرندیه با میانگین سنی 21/16 سال و انحراف معیار 45/1 به روش تصادفی چند مرحله‌ای انتخاب و وارد مطالعه شدند. ابزار جمع‌آوری اطلاعات یک پرسش‌نامه بی‌نام و طراحی شده بر اساس نظریه رفتار برنامه‌ریزی شده و هویت خود سیگار کشیدن بود که به روش خودگزارشی توسط نوجوانان تکمیل شد. اطلاعات جمع‌آوری شده با استفاده از نرم‌افزار Lisrel نسخه 8/8 و SPSS تجزیه و تحلیل شد. یافته‌ها: نظریه رفتار برنامه‌ریزی شده [معیار اطلاعات Akaike (Akaike information criterion یا AIC) 87/31، شاخص پراش بازدوگان 57/97 و شاخص مورد انتظار برای بررسی روایی 08/0) از برازش بهتری نسبت به نسخه نظریه رفتار برنامه‌ریزی شده توسعه یافته با هویت خود (معیار اطلاعات Akaike 42/54، شاخص پراش بازدوگان 77/176 و شاخص مورد انتظار برای بررسی روایی 15/0) برخودار بود. همچنین 52 درصد از واریانس قصد و 37 درصد از واریانس رفتار توسط نظریه رفتار برنامه‌ریزی شده توسعه یافته با هویت خود تبیین می‌شد که این میزان در نظریه رفتار برنامه‌ریزی شده به ترتیب 52 و 35 درصد بود. نتیجه‌گیری: نتایج این مطالعه از اضافه کردن سازه هویت خود به نظریه رفتار برنامه‌ریزی شده در رفتار مصرف سیگار حمایت نکرد. به علاوه در پیشگویی قصد رفتاری، کنترل رفتاری درک شده نقش مهم‌تری از سایر اجزای نظریه رفتار برنامه‌ریزی شده بازی می‌کند. در طراحی مداخلات مناسب‌سازی شده برای پیشگیری از مصرف دخانیات این موضوع باید مد نظر قرار گیرد.

Title: بررسی شیوع و عوامل مرتبط با پرخاشگری در بین نوجوانان شهر یاسوج

http://hsr.mui.ac.ir/index.php/jhsr/article/view/973

مقدمه: رفتار پرخاشگرانه دوران نوجوانی می‌تواند به عنوان یک عامل پیش‌بینی کننده برای بزهکاری، سوء مصرف مواد، افسردگی و افت تحصیلی باشد. این مطالعه با هدف تعیین شیوع و عوامل مرتبط با پرخاشگری در بین نوجوانان انجام شد. روش‌ها: پژوهش حاظر یک مطالعه توصیفی- مقطعی بود که در بین نوجوانان شهر یاسوج در سال 1389 صورت گرفت. آزمودنی‌های مطالعه به روش نمونه‌گیری تصادفی ساده از میادین اصلی در سطح شهر انتخاب شدند. ابزار جمع‌آوری اطلاعات، پرسش‌نامه‌ای شامل دو بخش اطلاعات دموگرافیک و پرسش‌نامه استاندارد پرخاشگری Boss و Perry بود، که به صورت خودگزارش‌دهی توسط شرکت کنندگان تکمیل گردید. اطلاعات به وسیله نرم‌افزار SPSS نسخه 18 مورد تجزیه و تحلیل قرار گرفت. یافته‌ها: شیوع پرخاشگری در بین نوجوانان 2/32 درصد بود. مصرف مواد مخدر، مصرف مشروبات الکلی، رابطه جنسی پرخطر، مصرف مواد مخدر در دوستان و طلاق والدین دارای رابطه آماری معنی‌دار با بروز پرخاشگری در نوجوانان بود (05/0 > P). نتیجه‌گیری: با توجه به شیوع بالای پرخاشگری، ارایه برنامه‌های آموزشی در خصوص ارتقای مهارت‌های کنترل خشم و پیشگیری از پرخاشگری در نوجوانان ضروری به نظر می‌رسد.

Title: بررسی دانش کارشناسان وزارت جهاد کشاورزی نسبت به کشاورزی ارگانیک

http://hsr.mui.ac.ir/index.php/jhsr/article/view/1116

مقدمه: كشاورزي ارگانيك در ميان تمام شيوه‌هاي مختلف، يكي از روش‌هايي است كه از محيط زيست محافظت مي‌كند. با توجه به نقش مهم کارشناسان کشاورزی در زمینه ترویج روش‌های کشاورزی ارگانیک و اهمیت میزان دانش کارشناسان نسبت به کشاورزی ارگانیک، تحقیق حاضر با هدف بررسی دانش کارشناسان ستادی وزارت جهاد کشاورزی نسبت به کشاورزی ارگانیک در سال 1390 انجام گردید. روش‌ها: مطالعه حاضر از نوع توصیفی- همبستگی به روش مقطعی بود که با روش نمونه‌گیری تصادفی طبقه‌ای که حجم آن با استفاده از جدول Cochran 154 نفر به دست آمد بر روی کارشناسان شاغل در وزارت جهاد کشاورزی انجام شد. برای جمع‌آوری داده‌ها از پرسش‌نامه محقق ساخته استفاده گردید که روایی ظاهری و محتوایی و پایایی پرسش‌نامه مورد تأیید قرار گرفت و اطلاعات به دست آمده به وسیله نرم‌افزار SPSS نسخه 19 مورد تجزیه و تحلیل قرار گرفت. یافته‌ها: میانگین سنی کارشناسان 44 سال که جوان‌ترین آن‌ها 30 سال و مسن‌ترین آن‌ها 60 ساله بود. کارشناسان در مجموع در مورد مفهوم و اهداف كشاورزي ارگانيك، دانشی در حد متوسط و نسبت به اصول کشاورزی ارگانیک و اصل سلامت، دانشی در حد بالا داشته‌اند. بین دانش کارشناسان و تعداد دوره‌های آموزش ضمن خدمت همبستگی مثبت و معنی‌داری وجود داشت.  نتیجه‌گیری: نتایج بیانگر دانش متوسط کارشناسان نسبت به کشاورزی ارگانیک می‌باشد. یافته‌ها نشان می‌دهند که کارشناسان پیرامون مقوله‌های عام، دارای دانش بالاتری نسبت به مقوله‌های خاص و تخصصی‌تر هستند. این می‌تواند زمینه‌ای برای برنامه‌ریزی‌های آموزشی هرچه بهتر دست‌اندرکاران این موضوع باشد. با توجه به یافته‌های بررسی همبستگی، دوره‌های آموزشی ضمن خدمت سبب افزایش سطح دانش کارشناسان نسبت به کشاورزی ارگانیک می‌شود و این نیز در امر برنامه‌ریزی‌های آموزشی بایستی لحاظ گردد.
